# Temperature‐dependent differences in male and female life history responses to a period of food limitation during development

**DOI:** 10.1111/1365-2656.70037

**Published:** 2025-03-24

**Authors:** Diego Moura‐Campos, Meng‐Han Joseph Chung, Edward Lawrence, Michael D. Jennions, Megan L. Head

**Affiliations:** ^1^ Division of Ecology and Evolution Australian National University, Research School of Biology Canberra Australian Capital Territory Australia; ^2^ Centre for Conservation Ecology and Genomics, Institute for Applied Ecology, Faculty of Science and Technology, University of Canberra Canberra Australian Capital Territory Australia

**Keywords:** catch‐up growth, environmental change, fish, Poecilidae, sex differences, sexual size dimorphism

## Abstract

1. With climate change, animals face both rising temperatures and more variable food availability. Many species have evolved an adaptative response to historic variation in food availability: they grow faster after a period of diet restriction (“compensatory growth”). However, higher temperatures may reduce the capacity for compensatory growth in ectotherms because individuals require more resources to support their increased metabolism.
2. We experimentally tested how higher temperature affects compensatory growth by raising guppies (Poecilia reticulata) at a high or control temperature, and on a normal or temporarily restricted diet during early development.
3. At the control temperature guppies on the restricted diet grew faster once their diet returned to normal. Both sexes showed compensatory growth. At the high temperature, both sexes also increased their growth rates after dietary restriction ended, but the life history outcomes differed. Males at the high temperature matured earlier and were smaller than males reared at the control temperature. In contrast, females at the high temperature matured later and were bigger than females at the control temperature.
4. Our study highlights that males and females can have different responses to the same environmental stressors.

## Graphical abstract

Our work highlights that high temperatures will not prevent guppies from exhibiting compensatory growth responses; however, development in warmer climates will lead to sex‐specific patterns in life‐history traits.

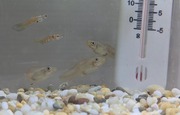

## 1 INTRODUCTION

Early environmental conditions play an important role in shaping animal development (Eyck et al., 2019; Imsland et al., 2020). During development, animals need to balance food acquisition between allocation to body maintenance and growth (Hou et al., 2008). However, natural variation in prey availability, population density and predation risk can all affect the ability of animals to acquire food during early development (Buskirk & Yurewicz, 1998; Garcia et al., 2018; Lima & Dill, 1990). Reduced food intake can lower the allocation of resources towards growth resulting in smaller adults that tend to have a higher rate of mortality and lower reproductive success (Blanckenhorn, 2000; Schindler et al., 2020). To minimise the impact of reduced food intake during development, many species have evolved an adaptive physiological response whereby they grow faster after a period of low food acquisition (so‐called ‘compensatory growth’).

Compensatory growth occurs in many taxa that have a wide range of life‐history strategies (Hector & Nakagawa, 2012). Compensatory growth can be achieved through an increased feeding rate or through physiological changes that improve the efficiency of food conversion (Hornick et al., 2000). By accelerating growth after a period of diet restriction, individuals can reach a similar size to age‐matched conspecifics that have not experienced a restricted diet (i.e. full compensatory growth). Full compensatory growth is particularly beneficial when time constraints (e.g. growth ceases at maturity) or intense sexual competition favours larger size at maturation (Dahl et al., 2012; Hector & Nakagawa, 2012; Orizaola et al., 2014). However, compensatory growth can have costs for some components of fitness. For instance, faster growth can impose short‐term ecological and physiological costs (e.g. greater exposure to predators and more oxidative damage during development) (Janssens & Stoks, 2020; Metcalfe & Monaghan, 2003; Stoks et al., 2006), as well as long‐term ‘hidden’ costs, such as reduced adult mobility, cognition, reproduction and lifespan (Almeida et al., 2021; Dahl et al., 2012; DeBlock & Stoks, 2008; Eyck et al., 2019; Metcalfe & Monaghan, 2003). Compensatory growth has been well documented (Hector & Nakagawa, 2012; Hornick et al., 2000), but it remains unclear how it is affected by environmental factors that influence bioenergetic trade‐offs, most notably temperature (Eyck et al., 2019).

Temperature is a key factor affecting developmental growth in ectotherms (Ritchie & Friesen, 2022). Higher temperatures generally increase metabolic rates, which tends to accelerate development (Brown et al., 2004; van der Have & de Jong, 1996). Ectotherms developing at warmer temperatures usually mature faster and tend to be smaller (Angilletta et al., 2004; Hoefnagel & Verberk, 2015; van der Have & de Jong, 1996) (the ‘temperature‐size rule’ (Atkinson, 1996)). In addition, higher temperatures can increase the costs of somatic maintenance due to faster accumulation of oxidative damage (Zhang et al., 2023). Consequently, organisms that experience both high temperatures and limited access to food during development are likely to face an increased challenge to allocate resources to allow for compensatory growth while still ensuring effective somatic maintenance (Eyck et al., 2019). High temperatures are therefore expected to exacerbate the costs of compensatory growth (Dinh et al., 2016; Grunst et al., 2023; Hardison & Eliason, 2024).

Growth patterns have been extensively studied in aquatic organisms, including fish, because of the economic implications (Ali et al., 2003; Mommsen, 2001; Py et al., 2022). Several studies show that fish exhibit full or partial compensatory growth after a period of diet restriction (reviewed in Py et al., 2022). Partial compensatory growth refers to cases where there is faster growth after dietary restriction ends, but animals mature at a smaller size than those on an unrestricted diet. To date, only a few studies have investigated how higher temperatures affect compensatory growth. These studies have mostly focused on temperate species (Abdollahpour et al., 2022; Pennock et al., 2021; Poletto et al., 2018; Rodgers et al., 2019; van Dijk et al., 2005; Yoneda & Wright, 2005), which makes their relevance to tropical species uncertain.

Tropical fish live at temperatures near their upper thermal limit and are expected to be more sensitive to increased temperatures than temperate species that usually have a wider thermal tolerance range (Donelson et al., 2012; Tewksbury et al., 2008). Guppies (*Poecilia reticulata*; Family: Poecilidae) are a tropical fish species that have become a model system for studies in ecology and evolution (Houde, 1997). In guppies, low food availability during development can have long‐term effects on somatic and reproductive traits (Evans et al., 2023; Kolluru et al., 2006, 2009; Kolluru & Grether, 2005). Guppies also show compensatory growth after diet restriction during early development, leading to a later decline in fecundity (Auer et al., 2010). Guppies are therefore an ideal species to test if higher than normal temperatures lower the ability of tropical fish to exhibit compensatory growth after a poor start in life.

Here we test if a higher temperature affects the response to diet restriction by guppies (*P. reticulata*). In a 2 × 2 experiment, we reared fish to maturation at either 26° or 30°C (air temperature; for water temperature see Section 2) and we manipulated juvenile food availability (control or restricted diet for 2 weeks). We measured initial and overall juvenile growth, time to maturation, and size at maturation. We discriminate between initial growth shortly after a period of food restriction ends and longer‐term growth to maturity. Faster initial growth is used to determine whether compensatory growth occurred. Based on studies of other poecilids (Auer, 2010; Auer et al., 2010; Livingston et al., 2014; Reznick, 1990; Vega‐Trejo et al., 2016), we predicted that:

1. At the control temperature (26°C), fish will show compensatory growth after 2 weeks of diet restriction. At the high temperature (30°C), however, fish will not show compensatory growth after 2 weeks of diet restriction. We make this prediction because dietary stress under high temperature is expected to lead to greater depletion of energy reserves, forcing fish to direct resources towards somatic maintenance instead of growth (Ali et al., 2003; Biro et al., 2007).
2. For fish on the control diet, those at the higher temperature will mature earlier and will be smaller than those at the control temperature. This is a common response to increased temperature in ectotherms (Atkinson, 1996).
3. For fish on the restricted diet for 2 weeks, those at the higher temperatures will mature later but will be smaller than fish on the restricted diet at the control temperature because increased metabolic costs at a higher temperature reduce the energy/resources available for growth (Eyck et al., 2019).
4. The size difference at maturity between fish on the control and restricted diet will be greater at the higher than at the control temperature since high energetic demands in high temperatures, along with reduced energy intake when diet restricted, may have additive long‐term effects on size.

## 2 METHODS

### 2.1 Origin and maintenance of fish

Guppy stocks are from two independent collections from an invasive population in Alligator Creek near Townsville, Australia (Kranz et al., 2018; Lindholm et al., 2014). Since 2019, these stocks have been kept in mixed‐sex tanks (~50 fish per 60 L aquaria) in controlled temperature rooms at 26 ± 1°C with a 14:10 light/dark cycle and fed twice daily with live brine shrimp nauplii (*Artemia* sp.) and fish flakes (Aqua One). To start our experiment, we collected newborn fry and transferred them into 7 L plastic tanks (up to 10 fry/tank). Juveniles were then inspected weekly to determine their sex. Immature males and females were transferred to single‐sex aquaria to ensure their virginity as adults. All animal care and procedures were approved by the ANU Animal Ethics committee (permit A2021/04).

### 2.2 Experimental protocol

To generate test fish, we randomly paired a virgin male and a virgin female in an individual 7 L tank (*n* = 28 pairs), under the same conditions as stock fish. After 2 weeks, we removed the males and then inspected the tanks daily for newborn fry. On the day of birth (Day 0), we transferred each offspring to its own 1 L tank. Newborn fry within each brood were alternately assigned to our four experimental treatments: control temperature/control diet; control temperature/restricted diet; high temperature/control diet; high temperature/restricted diet.

We manipulated temperature by placing tanks in four temperature‐controlled rooms (two rooms at each temperature) at either 26 ± 1°C (control) or 30 ± 1°C (high) on Day 0. This equated to water temperatures of 24.4 ± 0.6°C (control) and 27.8 ± 0.4°C (high). Water temperature in guppies' native habitat ranges from 20°C to 28°C (Alkins‐Koo, 2000), but the upper value is predicted to increase as global temperatures rise (Breckels & Neff, 2013; IPCC, 2023). Fish remained in their temperature treatment throughout the experiment. All rooms were virtually identical in size and environmental conditions, apart from the manipulated variable—temperature. Access to the rooms during the experiment was limited to the feeders and researchers. Newborn fry were fed ad libitum with brine shrimp nauplii for 3 days (Days 0–2) and then began their diet treatment on Day 3. Fry on the control diet were fed brine shrimp nauplii ad libitum (approximately 6 mg) twice daily, whereas fry on the restricted diet were fed 3 mg of brine shrimp once a day every second day for 14 days (i.e. 12.5% of control diet until day 17). This feeding regime led to near zero growth without elevated mortality in another poecilid fish (Vega‐Trejo et al., 2016). On Day 17, all fish were returned to the control diet. In total, we used offspring from 28 pairs to set up 110 fish per treatment (total *N* = 440).

To measure growth, we photographed fish on Day 3 (prior to diet restriction), Day 17 (at the end of diet restriction) and Day 31 (2 weeks after a return to the control diet). We placed each fry in a small container of shallow water containing a 1 cm ruler and then photographed it from above using a digital camera (Canon PowerShot SX620 HS). Standard length (tip of the snout to the end of the caudal peduncle) was later measured using *ImageJ* software (Rasband, 1997).

We inspected fish twice a week from Day 31 to determine the time to sexual maturity. We considered females to be sexually mature when we observed a visible, yellow egg spot near their rounded anal fin (Figure S1; Stearns, 1983; Zajitschek & Brooks, 2010; Livingston et al., 2014). The egg spot is visible when there is yolk formation inside the female body, which is the last stage of egg maturation in female guppies (Rodd & Reznick, 1997). Following Reznick (1990) and Houde (1997), males were considered to be mature when we observed a fully developed hood and hooks at the tip of their gonopodium. On the day of maturity, we anaesthetised each fish using AQUI‐S (New Zealand; 20 mg/L) for 30 seconds, placed it on its side on a glass slide, and photographed it next to a scaled ruler. Standard length was again measured using *ImageJ*.

We calculated the instantaneous rate of growth for three periods: (1) Day 3 to 17 (diet treatment); (2) Day 17 to 31; and (3) from the end of the diet restriction to sexual maturity (from Day 3 for fish on the control diet; from Day 17 for fish on the restricted diet). We used the formula:

$$G=\frac{\ln\;\left(\frac{\mathrm{Lt}1}{\mathrm{Lt}0}\right)}{t},$$

where *Lt*0 and *Lt*1 are lengths on the initial and final day of the focal period, respectively, and *t* is the duration of the period (in days) (Vega‐Trejo et al., 2016). We used *initial* growth to determine whether there is compensatory growth. *Initial growth* refers to the period immediately after the diet restriction ended (Days 17–31), or from Days 3–17 for control fish. This allows for a direct comparison across treatments for fish that were initially the same size. In a previous study, Vega‐Trejo et al. (2016) showed that the diet restriction treatment we used resulted in minimal growth such that Day 3 fish on the control diet and Day 17 fish on the restricted diet were almost identical in size. Indeed, in our study, body size was similar for restricted diet (Day 17) and control diet fish (Day 3) at the control temperature (control diet: mean (± SD): 11.40 ± 0.85 mm; restricted diet: mean (± SD): 11.61 ± 0.75 mm) and the high temperature (control diet: mean (± SD): 12.46 ± 0.93 mm; restricted diet: mean (± SD): 12.38 ± 0.84 mm). To test for compensatory growth, we therefore tested whether growth from Day 17–31 for fish on the restricted diet differed from growth from Day 3–17 for fish on the control diet. In addition, we quantified *overall* growth, which was defined as growth from the end of diet restriction (or its equivalent) until sexual maturity (i.e. starting at Day 3 for control fish and Day 17 for diet‐restricted fish). The mean duration of the growth period varied among the four treatments because the treatments affected the time to sexual maturation (see Section 3). Because the duration over which growth was measured differs among fish, it is more difficult to determine whether there is compensatory growth, because growth rates change with size, hence age.

### 2.3 Statistical analyses

The effects of temperature and diet restriction on our five focal traits (body size on Day 17; initial growth rate; overall growth rate; size at maturity; age at maturity) were analysed using individual linear mixed models (LMM) in the ‘lmer’ function of the ‘lme4’ package in R version 4.2.2 (R Core Team, 2022). To test whether fish exhibited compensatory growth in response to a restricted diet early in life, and whether compensatory growth was affected by temperature, we included early life diet (control or restricted), temperature (control or high) and their interaction as fixed effects in our models. Additionally, we included sex (male or female) and all possible two‐ and three‐way interactions involving sex in our models. Brood identity was included as a random factor to account for measurement of several offspring from the same family. We used histograms and Q‐Q plots to confirm that model residuals met assumptions of normality.

We removed any non‐significant higher order interactions to interpret lower order interactions and/or main effects (Engqvist, 2005). If our analysis revealed a significant three‐way or two‐way interaction involving sex, we ran separate analyses for each sex. If there was a significant interaction between diet and temperature, we conducted Tukey's post‐hoc pairwise comparisons (‘emmeans’ function of the ‘emmeans’ package) between the four treatment groups.

We used the ‘Anova’ function of the ‘car’ package (type III Wald chi‐square or F‐tests) to determine the *p*‐value (alpha = 0.05; two‐tailed). We pre‐registered all statistical analyses with the Open Science Framework (https://doi.org/10.17605/OSF.IO/X4MJW), and any deviations from our analyses are detailed in the Supporting Information.

## 3 RESULTS

The full and reduced models and all Tukey's post‐hoc pairwise comparisons are available in the Supporting Information (Tables S1–S16).

### 3.1 Body size after the period of diet restriction

There was no effect of sex on body size on Day 17 (sex: *χ* ^2^ = 0.959, *p* = 0.327; sex × diet: *χ* ^2^ = 0.416, *p* = 0.519; sex × temp: *χ* ^2^ = 0.298, *p* = 0.585). Diet and temperature interacted to affect body size at the end of the diet treatment on Day 17 (*χ* ^2^ = 99.858, *p* < 0.001). Control fish were 3.6 mm larger than diet‐restricted fish at the control temperature (Tukey's test, *p* < 0.001), and 4.8 mm larger at the high temperature (Tukey's test, *p* < 0.001). Fish on the control diet reached a significantly larger size on Day 17 when they were at the higher temperature (Tukey's test, *p* < 0.001; Figure S2). Fish on a restricted diet did not differ in body size at the high or control temperature (Tukey's test, *p* = 0.342; Figure S2).

### 3.2 Initial growth

**FIGURE 1:**
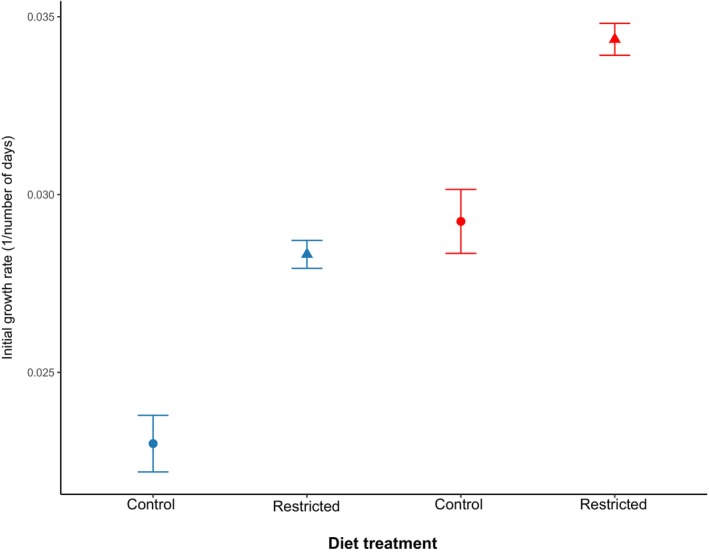
Mean initial growth rates (*G* = (ln(*Lt*1/*Lt*0))/*t*) separated by treatment (diet × temperature). Initial growth rates were measured from Days 3–17 for fish on the control diet (circles) and from Days 17–31 for fish that were diet restricted for the previous 2 weeks (triangles). The control temperature is represented in blue and the high temperature in red. Error bars are standard errors.

Sex had no effect on initial growth (three‐way interaction: *χ* ^2^ = 0.869, *p* = 0.351; sex × diet: *χ* ^2^ = 0.900, *p* = 0.340; sex × temp: *χ* ^2^ = 1.840, *p* = 0.170; sex: *χ* ^2^ = 0.715, *p* = 0.398; Table S3). Diet and temperature did not interact to affect initial growth (*χ* ^2^ = 0.900, *p* = 0.340; Figure 1). However, both diet and temperature independently affected initial growth. Initial growth is defined as the 14‐day period where initially similar‐sized fish had access to ad libitum food to grow (i.e. Days 3–17 for control fish and Days 17–31 for diet‐restricted fish). This means that immediately after the diet restriction period, fish on the restricted diet grew significantly faster for the next 2 weeks (Days 17–31) than initially similar‐sized fish on the control diet (days 3–17; *χ* ^2^ = 87.452, *p* < 0.001). There is therefore compensatory growth. Fish grew significantly faster at the high than at the control temperature (*χ* ^2^ = 119.213, *p* < 0.001).

### 3.3 Overall growth

Diet, temperature and sex interacted to affect overall growth (*χ* ^2^ = 5.919, *p* = 0.015). We therefore analysed each sex separately.

**FIGURE 2:**
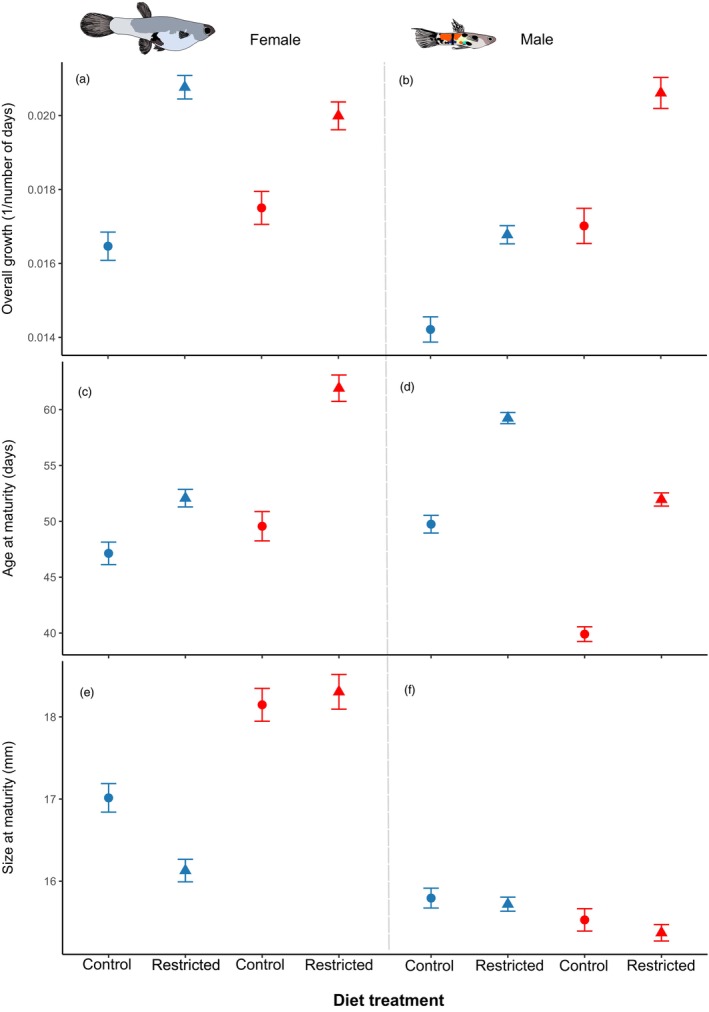
Mean overall growth rates (*G* = (ln(*Lt*1/*Lt*0))/*t*; a, b), age at maturity (c, d) and size at maturity (e, f) separated by sex (females: left; males: right) and treatment (diet × temperature). The control temperature is represented in blue and the high temperature in red. Circles represent the control diet and triangles represent the restricted diet during early life. Error bars are standard errors.

For females, there was a strong effect of diet on overall growth (*χ* ^2^ = 75.125, *p* < 0.001), with a weak interaction between diet and temperature (*χ* ^2^ = 4.195, *p* = 0.041). The strong effect of diet on overall growth meant that females that were initially on the restricted diet had greater overall growth than females that were on the control diet (Figure 2a). The weak interaction occurred because the difference in growth between the diets was slightly smaller at the high than at the control temperature. It should be noted, however, that the effect of temperature was not significant for fish on either the control or restricted diet (Tukey's test, *p* = 0.301 and *p* = 0.659, respectively), which is why we report the main effect of diet.

For males there was no interaction between diet and temperature (*χ* ^2^ = 2.1821, *p* = 0.140). Diet restriction (*χ* ^2^ = 72.0101, *p* < 0.001) and high temperature (*χ* ^2^ = 79.9817, *p* < 0.001) both lead to a significant increase in the overall growth rate (Figure 2b).

### 3.4 Age at maturity

There was no significant three‐way interaction between diet, temperature and sex (*χ* ^2^ = 2.918, *p* = 0.088), but there was an interaction between temperature and sex (*χ* ^2^ = 134.265, *p* < 0.001). We therefore analysed the sexes separately.

For females, diet and temperature interacted to affect age at maturity (*χ* ^2^ = 11.823, *p* < 0.001; Figure 2c). Females on the restricted diet matured significantly later at both the high and control temperature (Tukey's test, *p* = 0.005 and *p* < 0.001 respectively), but the difference was much greater at the high temperature (4.9 days vs. 12.4 days; Figure 2c).

For males, diet and temperature weakly interacted to affect age at maturity (*χ* ^2^ = 29.595, *p* < 0.033). At both the control and high temperature, males on the restricted diet matured significantly later than control fish, but the effect was slightly stronger at the high temperature (Tukey's tests, both *p* < 0.001; Figure 2d). Males also matured significantly sooner at the high temperature (Tukey's test, *p* < 0.001). The combined effect meant that the time to reach maturity for males on a restricted diet at the high temperature did not differ from males on the control diet at the control temperature (Tukey's test: *p* = 0.108; Figure 2d).

### 3.5 Size at maturity

Size at maturity was affected by a three‐way interaction between diet, temperature and sex (*χ* ^2^ = 5.389, *p* = 0.020). We therefore analysed the sexes separately.

For females, there was a significant interaction between diet and temperature that affected size at maturity (*χ* ^2^ = 9.108, *p* = 0.003). At the control temperature, females on the restricted diet matured at a smaller size than those on the control diet (Tukey's test, *p* = < 0.001; Figure 2e). But at the high temperature, females on the restricted and control diet matured at the same size (Tukey's test, *p* = 0.989; Figure 2e).

For males, there was no interaction between diet and temperature affecting size at maturity (*χ* ^2^ = 0.246, *p* = 0.620). There was also no effect of diet (*χ* ^2^ = 2.915, *p* = 0.088; Figure 2f). However, males matured at a smaller size at the high temperature than at the control temperature (*χ* ^2^ = 13.878, *p* < 0.001; Figure 2f).

## 4 DISCUSSION

Compensatory growth allows animals to reduce costs associated with smaller adult size and/or delayed maturation that would otherwise result after periods of diet restriction during development. While compensatory growth is commonplace in many taxa (Hector & Nakagawa, 2012), little is known about how environmental factors shape this diet‐mediated growth response. We investigated whether a higher temperature reduces an individual's ability to accelerate growth after a period of diet restriction that slowed its development. We found that the initial growth of guppies immediately after experiencing a restricted early‐life diet was faster than that of fish on a constant control diet. There was clear evidence for compensatory growth at both the control and high temperature. This did not support our prediction that compensatory growth would not occur at the higher temperature (see Prediction 1). A higher temperature did, however, alter the age and size at which guppies reached maturity, but did so differently for males and females. The response of males to the diet and temperature treatments supported two of our three predictions about time to maturity and size at maturity (Predictions 2–3; but not Prediction 4). In contrast, the response of females to diet and temperature treatments did not support our predictions about time to maturity and size at maturity (except for the time to maturity on the restricted diet). Our results highlight the need to test for sex differences when evaluating how temperature affects key life‐history traits.

### 4.1 Guppies show compensatory growth even at higher temperatures

At the control temperature, males and females showed a faster initial growth rate after a period of diet restriction than those on the control diet (i.e. compensatory growth). Contrary to our predictions, however, high temperature did not prevent guppies from increasing their initial or overall growth after diet restriction. This highlights that guppies exhibit compensatory growth immediately after the diet restriction period and maintain higher growth until sexual maturity. This faster growth occurred even at a higher temperature where energetic demands are higher (Ritchie & Friesen, 2022). Interestingly, absolute growth was greater at the higher temperature. This highlights that even when there is compensatory growth at the control temperature, guppies were not growing at their maximum rate. Under natural conditions, there may be selection against maximising growth either because of immediate ecological costs, such as predation associated with foraging, or because of hidden fitness costs that arise later in life (Dmitriew, 2011). Our results suggest that selection on growth changes with temperature since a higher temperature increased growth rates, which—all else being equal—should increase ecological and/or physiological costs. Our experimental design allowed animals to acclimate to high temperatures from birth, which might lower the short‐term physiological costs of generating reactive oxygen species during growth (Ritchie & Friesen, 2022). This could explain how guppies could sustain faster growth at high temperatures. Future research should explore how fluctuating temperatures affect compensatory growth since continued acclimatisation to new temperatures is expected to impose high physiological costs on ectotherms (Ritchie & Friesen, 2022), potentially hindering growth.

### 4.2 Sex differences in developmental patterns

At both temperatures, males showed full compensatory growth because those initially on a restricted diet reached maturity at the same size as their well‐fed counterparts. Overall, however, males reached maturity at a smaller size at the higher than at the control temperature. In contrast, females showed full compensatory growth at the high temperature, but only partial compensatory growth at the control temperature. Females that were initially on a restricted diet reached maturity at the same size as their well‐fed counterparts at the high temperature, but at a significantly smaller size than their well‐fed counterparts at the control temperature. These results broadly corroborate previous research on guppies showing full compensatory growth by males (Reznick, 1990) and partial compensatory growth by females (Auer, 2010).

Prey availability, population density and predation pressure all vary in freshwater ecosystems (Burns et al., 2009; Endler, 1995; Reznick et al., 2001) causing variation in food acquisition by guppies (Arendt & Reznick, 2005). It is likely that compensatory growth under natural conditions allows guppies to compensate for slower early growth and improve adult fitness (Auer, 2010). Compensatory growth is expected when there is strong selection for larger adults, either because size increases survival (Rodd & Reznick, 1997) or reproductive success (Ahti et al., 2020; Barneche et al., 2018). In guppies, both sexes prefer larger mates (Herdman et al., 2004; Magellan et al., 2005). Larger males also obtain advantages during male–male contests (Guerrera et al., 2022; Órfão et al., 2019); while larger females are more fecund (Barneche et al., 2018; Herdman et al., 2004; Holtby & Healey, 1990). Thus, both sexes appear to benefit from being larger. The sexes differ, however, in that males barely grow after they reach sexual maturity (Jordan & Brooks, 2010), while females continue to grow as adults. Consequently, there may be stronger selection on males against maturing at a smaller size. Why then do females show compensatory growth if there are potential long‐term costs? One possibility is that once females start to reproduce, those that fail to show compensatory growth after being on a restricted diet would never match the fecundity of their well‐fed counterparts. Alternatively, faster juvenile growth could be a byproduct of the greater food consumption that is commonly seen in fishes after dietary restriction ends (Ali et al., 2003; Hornick et al., 2000). In fish, this response is triggered upon re‐feeding by an increase in the production of the hunger‐inducing hormone ghrelin and neuropeptide Y (Won & Borski, 2013). Increased food consumption after food deprivation may have evolved to avoid starvation in environments with fluctuating food availability rather than for benefits associated with faster growth.

### 4.3 Developmental patterns will be modified as temperatures rise

Although the high temperature did not prevent compensatory growth, it had opposing effects on male and female development. Males matured at a smaller size at the high than control temperature, regardless of their diet. In contrast, females matured at a larger size at the high than control temperature, albeit with the effect being stronger for females on the restricted diet (Figure 2e,f). Faster development at higher temperatures is widely attributed to temperature elevating metabolic rates (Angilletta et al., 2004; Gopal et al., 2023), which implies that both sexes will respond similarly to rising temperatures. In support of this, high temperatures have been linked to a decrease in sexual size dimorphism in arthropods and some ectothermic vertebrates (arthropods: Hirst et al., 2015; Chelini et al., 2019; lizards: Starostová et al., 2010; amphibians: Angelini et al., 2015; reviewed in Badyaev, 2002). Most fishes, however, have sex differences in the time available for growth, where males largely stop growing after sexual maturity, while females continue to grow. This could explain the increase in sexual size dimorphism under high temperatures (Estlander et al., 2017; Rennie et al., 2008; van Dorst et al., 2024). It is plausible that the sexes do not respond in the same way because males and females optimise their life histories in different ways (Immonen et al., 2018; Parker, 1992). Larger females benefit by producing more eggs (Barneche et al., 2018; Herdman et al., 2004; Holtby & Healey, 1990), while males might gain a net benefit by offsetting costs of being smaller adults (Magellan et al., 2005; Niu et al., 2023) against the benefit that maturing sooner prolongs access to females (Angilletta et al., 2004; Blanckenhorn, 2000). Controlling for other factors, time to and size at maturity are usually negatively related in poecilids (Auer, 2010; Auer et al., 2018; Boulton et al., 2016; Vega‐Trejo et al., 2016). Intriguingly, the sexes differed in how treatment differences in the size at maturity were mediated by the time taken to reach maturity. Controlling for diet, at the higher temperature, males grew faster and reached maturity sooner. In contrast, at the higher temperature females on the restricted diet took far longer to reach maturity than females on the control diet but still ended up at the same size at maturity. This suggests that a restricted diet lowered the ability of females to grow after the initial phase of high compensatory growth.

We have discussed the effect of temperature on development, assuming the observed responses are adaptive. It is, however, worth noting that temperature can set limits on biological functions and impose physiological constraints. The observed temperature‐dependent changes in development might not be adaptive plastic responses (Angilletta et al., 2004). Our high temperature treatment was such that fish were closer to their upper thermal limit for an extended period, which is a situation outside the average historic conditions that guppies have experienced (Alkins‐Koo, 2000; Grinder et al., 2020). The observed growth and maturation patterns might therefore reflect physiological constraints. For example, high temperatures tend to increase feeding rates in fish (Hardison & Eliason, 2024; Horppila et al., 2011; Volkoff & Rønnestad, 2020), and their growth hormone production (Deane & Woo, 2009; Gabillard et al., 2005). Both these changes should accelerate growth and shorten the time to maturation. However, the sexes differed in their response to a higher temperature, so additional factors are required to invoke a non‐adaptive explanation. For example, there might be an interaction between higher growth hormone production at higher temperatures and sex‐specific steroids causing sex differences in the time to, and size at, maturation (Celino‐Brady et al., 2022; Mandiki et al., 2004; Rennie et al., 2008).

## 5 CONCLUSIONS

A high temperature did not directly affect compensatory growth, but it changed development in a sex‐specific manner: after diet restriction, males matured smaller and sooner, while females matured later and larger than their counterparts on the control diet. Compensatory growth is viewed as an adaptive response to a temporary reduction in food availability during development (Gagliano & McCormick, 2007), but studies have shown that faster growth can have long‐term fitness costs (e.g. Ali et al., 2003; Álvarez & Metcalfe, 2005; Py et al., 2022). Given that we found that the higher temperature increased growth rates and modified developmental patterns, rising temperatures could exacerbate existing fitness costs of compensatory growth reported for guppies (Auer et al., 2010). Future studies should investigate if early‐life diet restriction at higher temperatures is associated with a greater decline in fecundity, which could lower population growth or the likelihood of persistence. The global temperature is predicted to rise, and food availability to become more variable, making it important to understand their combined effects. This is especially the case for tropical fishes, like guppies, that are sensitive to temperature changes due to their lower thermal range and more complex trophic interactions (Donelson et al., 2012; Tewksbury et al., 2008).

## AUTHOR CONTRIBUTIONS

Diego Moura‐Campos, Meng‐Han Joseph Chung, Edward Lawrence and Megan L. Head designed the study. Diego Moura‐Campos, Meng‐Han Joseph Chung and Edward Lawrence conducted the experiment. Diego Moura‐Campos analysed the data and drafted the manuscript, with Megan L. Head, Meng‐Han Joseph Chung and Michael D. Jennions providing critical revisions. All authors revised and approved the final manuscript.

## CONFLICT OF INTEREST STATEMENT

The authors declare no conflicts of interest.

## Supporting information


**Data S1:** Supplementary methods.

## Data Availability

All data and R code have been deposited at Mendeley Data: https://data.mendeley.com/datasets/5sbwbcmndh/1 (Moura‐Campos et al., 2024).
