# Supplementary material for: Temperature‐dependent differences in male and female life history responses to a period of food limitation during development
Source: J Anim Ecol. 2025 Mar 24;94(5):1076–87. doi: 10.1111/1365-2656.70037 (PMC12056356; doi:10.1111/1365-2656.70037)
Supplement: Supplementary file 1 — Data S1: Supplementary methods. [file JANE-94-1076-s001.docx]

**Temperature-dependent differences in male and female life history responses to a period of food limitation during development**

Diego Moura-Campos^1^, Meng-Han Joseph Chung^1,2^, Edward Lawrence^1^, Michael D. Jennions^1^, Megan L. Head^1^

^1^Australian National University, Research School of Biology, Division of Ecology and Evolution, Acton, 2601, ACT, Australia

^2^Centre for Conservation Ecology and Genomics, Institute for Applied Ecology, Faculty of Science and Technology, University of Canberra, Canberra, Bruce ACT, 2617, Australia

**Supplementary materials**

**Supplementary methods**

Statistical analysis

We have pre-registered our statistical analyses following the Open Science Framework (https://doi.org/10.17605/OSF.IO/X4MJW). We have included another growth rate variable that was not present in our original analyses. For that, we calculated growth rate until sexual maturity for all treatments to better understand overall compensatory growth, following (Vega-Trejo et al., 2016). Additionally, we originally described performing model analysis including all three-way interactions (diet, temperature, and sex). Since we obtained multiple two and three-way interactions for several of our models and given the biological differences between male and female guppies, we extended our original analyses by splitting our models for three variables (overall growth rate, age, and size at maturity) by sex to better understand the sex specific effects.

References

Vega-Trejo, R., Head, M. L., & Jennions, M. D. (2016). Inbreeding depression does not increase after exposure to a stressful environment: A test using compensatory growth. *BMC Evolutionary Biology*, *16*(1), 68. https://doi.org/10.1186/s12862-016-0640-1

**Supplementary figures**

**
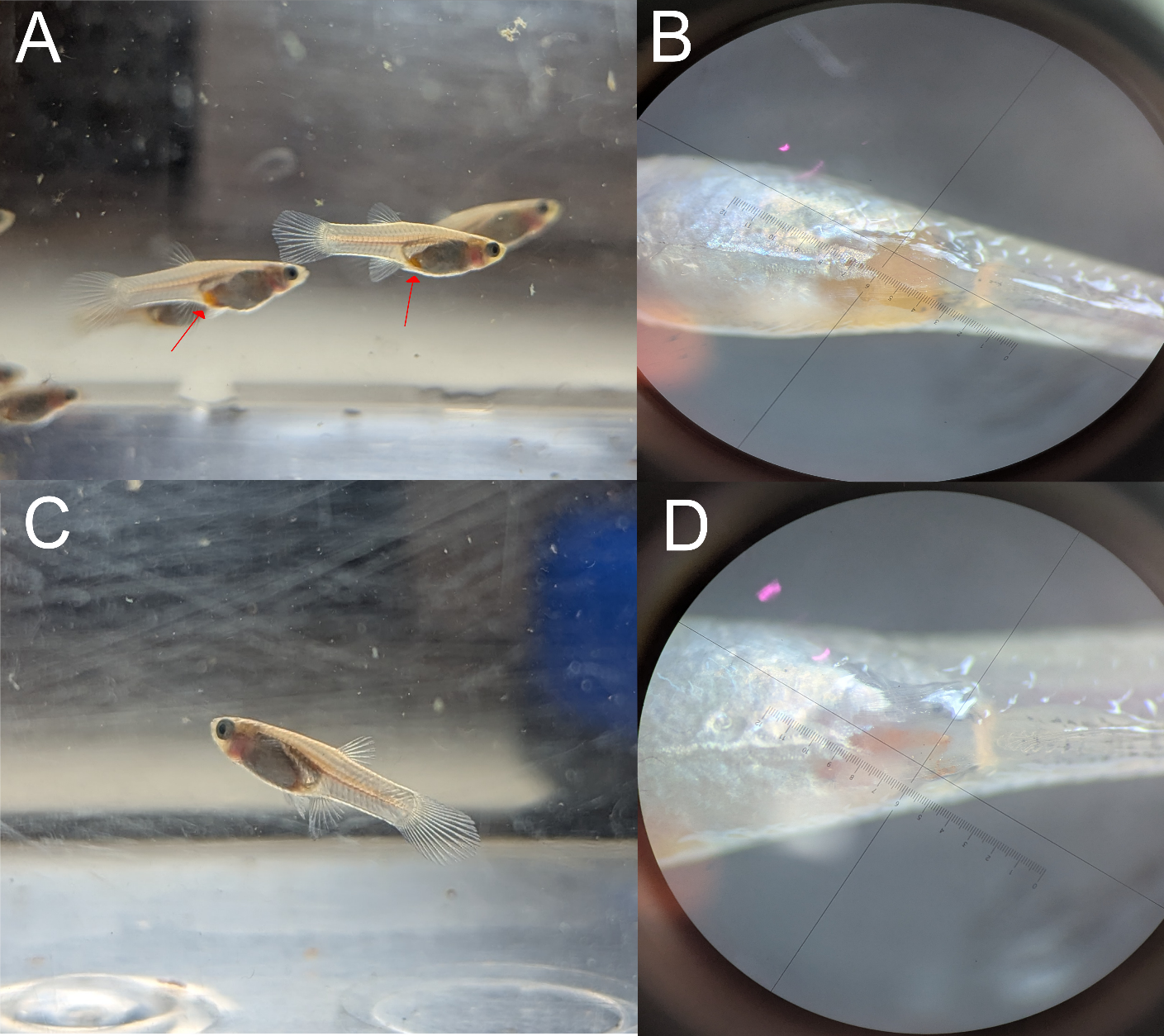
**

Supplementary Figure 1. Comparison of female guppies at different maturation stages. Female guppy at approximately 55 days of age showing a visible yellow egg spot near the anal fin in a lateral view opposite to a light source (A) and ventral view under a stereomicroscope (B). Female guppy at approximately 37 days of age (before reaching sexual maturity), shown in a lateral view opposite to a light source (C) and ventral view under a stereomicroscope (D).


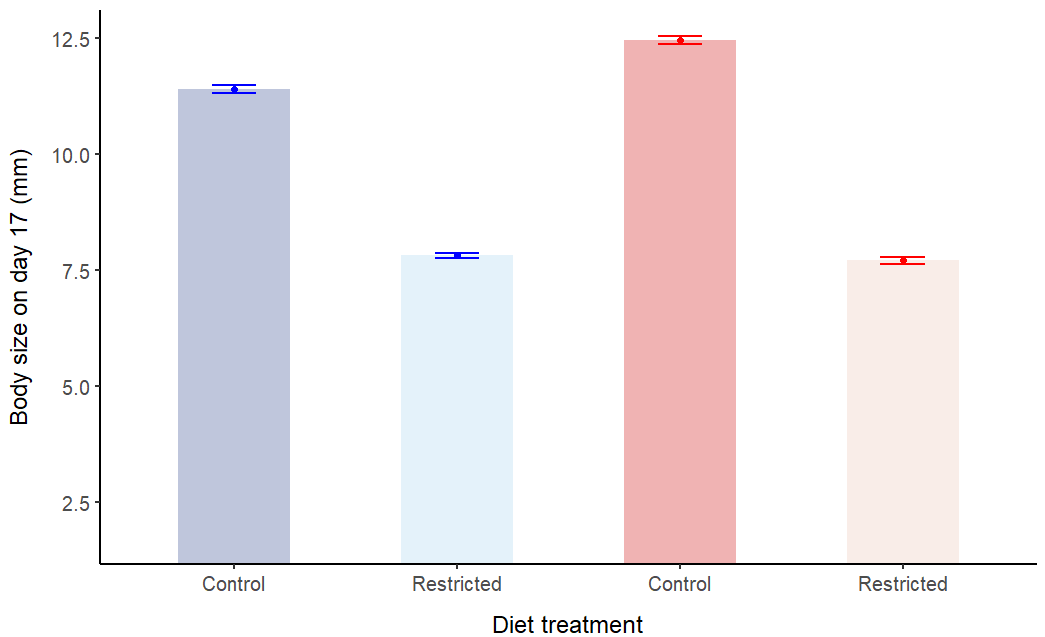


Supplementary Figure 2. Mean body size immediately after diet restriction period (day 17) separated by treatment (diet * temperature). The control temperature is represented in blue and the high temperature in red. Error bars are standard errors.

Supplementary Table 1. Results from linear mixed models (LMM) of body size at day 17 (after diet restriction period) with chi-square (χ2) values for significance tests of estimated parameters for diet, temperature, and sex.

| Full model | | | Estimate | *SE* | *df* | χ² | *P* |
| --- | --- | --- | --- | --- | --- | --- | --- |
| Fixed effects | |  |  |  |  |  |  |
| Intercept (control, control, female) | | 11.425 | 0.120 | 82.368 | 9034.556 | < 0.001 |  |
| Diet (restricted) | | -3.521 | 0.122 | 395.558 | 830.115 | < 0.001 |  |
| Temperature (high) | | 1.114 | 0.121 | 397.550 | 84.018 | < 0.001 |  |
| Sex (male) | | -0.004 | 0.124 | 398.162 | 0.001 | 0.974 |  |
| Diet * Temp | | -1.226 | 0.175 | 397.173 | 49.208 | < 0.001 |  |
| Diet * Sex | | -0.086 | 0.176 | 399.030 | 0.238 | 0.625 |  |
| Temp * Sex | | -0.074 | 0.179 | 401.508 | 0.168 | 0.682 |  |
| Diet * Temp * Sex | | 0.010 | 0.253 | 400.973 | 0.001 | 0.970 |  |
| Random effect | **Variance** | ***sd*** | **Number of groups** | |  |  |  |
| Brood ID (intercept) | 0.1889 | 0.4346 | 28 | |  |  |  |
| Two-way model | | | **Estimate** | ***SE*** | ***Df*** | **χ²** | ***P*** |
| Fixed effects | |  |  |  |  |  |  |
| Intercept (control, control, female) | | 11.426 | 0.117 | 74.130 | 9574.518 | **< 0.001** |  |
| Diet (restricted) | | -3.523 | 0.105 | 396.187 | 1109.192 | **< 0.001** |  |
| Temperature (high) | | 1.111 | 0.104 | 396.928 | 112.039 | **< 0.001** |  |
| Sex (male) | | -0.006 | 0.107 | 399.779 | 3.6e-3 | 0.952 |  |
| Temp * Sex | | -0.688 | 0.125 | 400.912 | 0.298 | 0.585 |  |
| Diet * Sex | | -0.081 | 0.125 | 401.5 | 0.416 | 0.519 |  |
| Diet * Temp | | -1.221 | 0.122 | 394.401 | 99.858 | **< 0.001** |  |
| Random effect | **Variance** | ***sd*** | **Number of groups** | |  |  |  |
| Brood ID (intercept) | 0.1889 | 0.4346 | 28 | |  |  |  |
| Reduced model | | | **Estimate** | ***SE*** | ***Df*** | **χ²** | ***P*** |
| Fixed effects | | |  |  |  |  |  |
| Intercept (control, control, female) | | | 11.76 | 0.11 | 55.17 | 11592.93 | **< 0.001** |
| Diet (restricted) | | | -4.16 | 0.07 | 399.75 | 3707.26 | **< 0.001** |
| Temperature (high) | | | 0.47 | 0.07 | 398.19 | 47.71 | **< 0.001** |
| Sex (male) | | | -0.07 | 0.07 | 404.56 | 0.959 | 0.327 |
| Random effect | | **Variance** | ***sd*** | **Number of groups** | |  |  |
| Brood ID (intercept) | | 0.186 | 0.4312 | 28 | |  |  |

Supplementary Table 2. Results from estimated marginal means (EEMs) pairwise comparisons among four treatments for body size at day 17 (after diet restriction period) with t-ratio values for significance tests.

| Contrast | t ratio |  | p value |
| --- | --- | --- | --- |
| Control diet, control temp - Restricted diet, control temp | 41.589 |  | **< 0.001** |
| Control diet, control temp - Control diet, high temp | -12.493 |  | **< 0.001** |
| Control diet, control temp - Restricted diet, high temp | 42.542 |  | **< 0.001** |
| Restricted diet, control temp - Control diet, high temp | -53.937 |  | **< 0.001** |
| Restricted diet, control temp - Restricted diet, high temp | 1.667 |  | 0.342 |
| Control diet, high temp - Restricted diet, high temp | 54.724 |  | **< 0.001** |

Supplementary Table 3. Results from linear mixed models (LMM) of initial growth rate with chi-square (χ2) values for significance tests of estimated parameters for diet, temperature, and sex.

| Full model | | | Estimate | | *SE* | | *df* | χ² | | *P* | |
| --- | --- | --- | --- | --- | --- | --- | --- | --- | --- | --- | --- |
| Fixed effects | |  | |  | |  |  | |  | |  |
| Intercept (control, control, female) | | 0.022 | | 0.001 | | 89.199 | 425.125 | | < 0.001 | |  |
| Diet (restricted) | | 0.006 | | 0.001 | | 387.933 | 32.197 | | < 0.001 | |  |
| Temperature (high) | | 0.008 | | 0.001 | | 389.022 | 43.627 | | < 0.001 | |  |
| Sex (male) | | 0.001 | | 0.001 | | 391.069 | 1.371 | | 0.242 | |  |
| Diet * Temp | | -0.001 | | 0.002 | | 388.874 | 0.493 | | 0.482 | |  |
| Diet * Sex | | -0.002 | | 0.002 | | 392.199 | 1.772 | | 0.183 | |  |
| Temp * Sex | | -0.003 | | 0.002 | | 394.563 | 2.607 | | 0.106 | |  |
| Diet * Temp * Sex | | 0.002 | | 0.002 | | 393.892 | 0.869 | | 0.351 | |  |
| Random effect | **Variance** | | ***sd*** | | **Number of groups** | | |  | |  | |
| Brood ID (intercept) | 1.274e-05 | | 0.003 | | 28 | | |  |  |  |  |
| Two-way interaction model | | | Estimate | | *SE* | | *df* | χ² | | *P* | |
| Fixed effects | |  | |  | |  |  | |  | |  |
| Intercept (control, control, female) | | 0.022 | | 0.001 | | 79.753 | 461.460 | | < 0.001 | |  |
| Diet (restricted) | | 0.006 | | 0.001 | | 388.336 | 36.010 | | < 0.001 | |  |
| Temperature (high) | | 0.007 | | 0.001 | | 388.861 | 50.590 | | < 0.001 | |  |
| Sex (male) | | 0.001 | | 0.001 | | 392.566 | 0.670 | | 0.410 | |  |
| Diet * Sex | | -0.001 | | 0.001 | | 394.390 | 0.900 | | 0.340 | |  |
| Temp * Sex | | -0.002 | | 0.001 | | 394.466 | 1.840 | | 0.170 | |  |
| Diet * Temp | | -5.8e-05 | | 0.001 | | 385.971 | 0.000 | | 0.960 | |  |
| Random effect | **Variance** | | ***sd*** | | **Number of groups** | | |  | |  | |
| Brood ID (intercept) | 1.281e-05 | | 0.003 | | 28 | | |  |  |  |  |
| Reduced model | | | **Estimate** | | ***SE*** | | ***df*** | **χ²** | | ***P*** | |
| Fixed effects | |  | |  | |  |  | |  | |  |
| Intercept (control, control, female) | | 0.023 | | 9.118e-04 | | 51.251 | 615.658 | | **< 0.001** | |  |
| Diet (restricted) | | 0.005 | | 5.713e-04 | | 390.951 | 87.452 | | **< 0.001** | |  |
| Temperature (high) | | 0.006 | | 5.711e-04 | | 389.373 | 119.213 | | **< 0.001** | |  |
| Sex (male) | | -4.942e-4 | | 5.843e-04 | | 396.244 | 0.715 | | 0.398 | |  |
| Random effect | **Variance** | | ***sd*** | | **Number of groups** | | |  |  |  |  |
| Brood ID (intercept) | 1.297e-05 | | 3.601e-3 | | 28 | | |  |  |  |  |

Supplementary Table 4. Results from linear mixed models (LMM) of overall growth rate with chi-square (χ2) values for significance tests of estimated parameters for diet, temperature, and sex. Given a significant three-way interaction, we followed this up by running separate models for males and females to look at sex specific effects (see Supplementary Tables 5 and 7).

| Full model | | | Estimate | *SE* | *df* | χ² | *P* |
| --- | --- | --- | --- | --- | --- | --- | --- |
| Fixed effects | |  |  |  |  |  |  |
| Intercept (control, control, female) | | 0.016 | 0.000 | 131.059 | 1689.546 | < 0.001 |  |
| Diet (restricted) | | 0.004 | 0.000 | 389.137 | 76.487 | **< 0.001** |  |
| Temperature (high) | | 0.001 | 0.000 | 391.428 | 4.317 | **0.038** |  |
| Sex (male) | | -0.002 | 0.000 | 393.368 | 25.195 | **< 0.001** |  |
| Diet * Temp | | -0.002 | 0.001 | 390.577 | 5.423 | **0.020** |  |
| Diet * Sex | | -0.001 | 0.001 | 394.761 | 4.619 | **0.032** |  |
| Temp * Sex | | 0.002 | 0.001 | 398.156 | 6.761 | **0.009** |  |
| Diet * Temp * Sex | | 0.002 | 0.001 | 396.665 | 5.919 | **0.015** |  |
| Random effect | **Variance** | ***sd*** | **Number of groups** | |  |  |  |
| Brood ID (intercept) | 1.255e-06 | 0.001 | 28 | |  |  |  |

Supplementary Table 5. Results from linear mixed models (LMM) of overall female growth rate with chi-square (χ2) values for significance tests of estimated parameters for diet and temperature.

| Full model | | Estimate | *SE* | *df* | | χ² | *P* |
| --- | --- | --- | --- | --- | --- | --- | --- |
| Fixed effects | |  |  |  | |  |  |
| Intercept (control, control) | | 0.017 | 4.213e-04 | 68.265 | | 1536.714 | < 0.001 |
| Diet (restricted) | | 0.004 | 4.666e-04 | 190.928 | | 75.125 | **< 0.001** |
| Temperature (high) | | 0.001 | 4.758e-04 | 192.370 | | 3.080 | 0.079 |
| Diet * Temp | | -0.001 | 6.822e-04 | 191.542 | | 4.195 | **0.041** |
| Random effect | **Variance** | ***sd*** | **Number of groups** | |  |  |  |
| Brood ID (intercept) | 1.766e-06 | 0.001 | 28 | |  |  |  |

Supplementary Table 6. Results from estimated marginal means (EEMs) pairwise comparisons among four treatments (diet * temperature) for overall female growth rate with t-ratio values for significance tests.

| Pairwise comparison | t ratio |  | p value |
| --- | --- | --- | --- |
| Control diet, control temp - Restricted diet, control temp | -8.65 |  | **< 0.001** |
| Control diet, control temp - Control diet, high temp | -1.751 |  | 0.301 |
| Control diet, control temp - Restricted diet, high temp | -7.263 |  | **< 0.001** |
| Restricted diet, control temp - Control diet, high temp | 6.646 |  | **< 0.001** |
| Restricted diet, control temp - Restricted diet, high temp | 1.15 |  | 0.659 |
| Control diet, high temp - Restricted diet, high temp | -5.345 |  | **< 0.001** |

Supplementary Table 7. Results from linear mixed models (LMM) of overall male growth rate with chi-square (χ2) values for significance tests of estimated parameters for diet and temperature.

| Full model | | | | Estimate | | *SE* | *df* | | χ² | *P* |
| --- | --- | --- | --- | --- | --- | --- | --- | --- | --- | --- |
| Fixed effects | | |  | |  |  | |  |  |  |
| Intercept (control, control) | | | 0.014 | | 4.142e-04 | 87.885 | | 1158.130 | < 0.001 |  |
| Diet (restricted) | | | 0.003 | | 4.898e-04 | 186.770 | | 26.462 | < 0.001 |  |
| Temperature (high) | | | 0.003 | | 5.137e-04 | 191.652 | | 26.399 | < 0.001 |  |
| Diet * Temp | | | 0.001 | | 7.104e-04 | 188.155 | | 2.182 | 0.140 |  |
| Random effect | **Variance** | ***sd*** | | **Number of groups** | | |  |  |  |  |
| Brood ID (intercept) | 1.103e-06 | 0.001 | | 28 | | |  |  |  |  |
| Reduced model | | | | **Estimate** | | ***SE*** | ***df*** | | **χ²** | ***P*** |
| Fixed effects | | |  | |  |  | |  |  |  |
| Intercept (control, control) | | | 0.014 | | 3.719e-04 | 65.24 | | 1382.316 | < 0.001 |  |
| Diet (restricted) | | | 0.003 | | 3.557e-04 | 189.9 | | 72.010 | **< 0.001** |  |
| Temperature (high) | | | 0.003 | | 3.565e-04 | 190.6 | | 79.982 | **< 0.001** |  |
| Random effect | **Variance** | ***sd*** | | **Number of groups** | | |  |  |  |  |
| Brood ID (intercept) | 1.086e-06 | 0.001 | | 28 | | |  |  |  |  |

Supplementary Table 8. Results from linear mixed models (LMM) of age at maturity with chi-square (χ2) values for significance tests of estimated parameters for diet, temperature, and sex. Given multiple two-way interactions, we followed this up by running separate models for males and females to look at sex specific effects (see Supplementary table 9 and 10).

| Full model | | | | Estimate | | *SE* | *df* | | χ² | *P* |
| --- | --- | --- | --- | --- | --- | --- | --- | --- | --- | --- |
| Fixed effects | | |  | |  |  | |  |  |  |
| Intercept (control, control, female) | | | 46.832 | | 0.916 | 231.241 | | 2611.571 | **< 0.001** |  |
| Diet (restricted) | | | 5.104 | | 1.222 | 406.064 | | 17.437 | **< 0.001** |  |
| Temperature (high) | | | 2.473 | | 1.212 | 410.701 | | 4.161 | **0.041** |  |
| Sex (male) | | | 2.922 | | 1.237 | 410.853 | | 5.580 | **0.018** |  |
| Diet * Temp | | | 7.215 | | 1.748 | 409.379 | | 17.034 | **< 0.001** |  |
| Diet * Sex | | | 4.107 | | 1.751 | 412.383 | | 5.500 | 0.019 |  |
| Temp * Sex | | | -12.431 | | 1.780 | 416.391 | | 48.751 | **< 0.001** |  |
| Diet * Temp * Sex | | | -4.306 | | 2.521 | 415.190 | | 2.918 | 0.088 |  |
| Random effect | **Variance** | ***sd*** | | **Number of groups** | | |  |  |  |  |
| Brood ID (intercept) | 3.055 | 1.748 | | 28 | | |  |  |  |  |
| Two-way model | | | | **Estimate** | | ***SE*** | ***Df*** | | **χ²** | ***P*** |
| Fixed effects | | |  | |  |  | |  |  |  |
| Intercept (control, control, female) | | | 46.349 | | 0.876 | 203.246 | | 2799.764 | **< 0.001** |  |
| Diet (restricted) | | | 6.140 | | 1.064 | 405.370 | | 33.312 | **< 0.001** |  |
| Temperature (high) | | | 3.497 | | 1.055 | 407.369 | | 10.982 | **0.001** |  |
| Sex (male) | | | 3.974 | | 1.076 | 411.618 | | 13.643 | **< 0.001** |  |
| Diet * Temp | | | 5.100 | | 1.236 | 400.699 | | 17.028 | **< 0.001** |  |
| Diet * Sex | | | 2.017 | | 1.257 | 414.764 | | 2.576 | 0.109 |  |
| Temp * Sex | | | -14.587 | | 1.259 | 415.115 | | 134.265 | **< 0.001** |  |
| Random effect | **Variance** | ***sd*** | | **Number of groups** | | |  |  |  |  |
| Brood ID (intercept) | 3.156 | 1.777 | | 28 | | |  |  |  |  |

Supplementary Table 9. Results from linear mixed models (LMM) of female age at maturity with chi-square (χ2) values for significance tests of estimated parameters for diet, temperature, and sex.

| Full model | | | | Estimate | *SE* | *df* | χ² | *P* |
| --- | --- | --- | --- | --- | --- | --- | --- | --- |
| Fixed effects | | |  |  |  |  |  |  |
| Intercept (control, control, female) | | | 46.839 | 1.145 | 106.537 | 1673.010 | < 0.001 |  |
| Diet (restricted) | | | 4.970 | 1.472 | 204.291 | 11.406 | **< 0.001** |  |
| Temperature (high) | | | 2.352 | 1.464 | 207.911 | 2.580 | 0.108 |  |
| Diet * Temp | | | 7.258 | 2.111 | 206.868 | 11.823 | **< 0.001** |  |
| Random effect | | **Variance** | ***sd*** | **Number of groups** | |  |  |  |
| Brood ID (intercept) | | 6.457 | 2.541 | 28 | |  |  |  |
| Reduced model | | | | Estimate | *SE* | *df* | χ² | *P* |
| Fixed effects | |  |  |  |  |  |  |  |
| Intercept (control, control, female) | | 45.192 | 1.061 | 83.114 | 1812.597 | < 0.001 |  |  |
| Diet (restricted) | | 8.524 | 1.075 | 206.317 | 62.852 | **< 0.001** |  |  |
| Temperature (high) | | 5.860 | 1.077 | 207.670 | 29.595 | **< 0.001** |  |  |
| Random effect | **Variance** | ***sd*** | **Number of groups** | |  |  |  |  |
| Brood ID (intercept) | 6.557 | 2.561 | 28 | |  |  |  |  |

Supplementary Table 10. Results from estimated marginal means (EEMs) pairwise comparisons among four treatments (diet * temperature) for female age at maturity with t-ratio values for significance tests.

| Contrast | t ratio |  | p value |
| --- | --- | --- | --- |
| Control diet, control temp - Restricted diet, control temp | -3.368 |  | **0.005** |
| Control diet, control temp - Control diet, high temp | -1.599 |  | 0.382 |
| Control diet, control temp - Restricted diet, high temp | -9.813 |  | **< 0.001** |
| Restricted diet, control temp - Control diet, high temp | 1.754 |  | 0.299 |
| Restricted diet, control temp - Restricted diet, high temp | -6.328 |  | **< 0.001** |
| Control diet, high temp - Restricted diet, high temp | -8.096 |  | **< 0.001** |

Supplementary Table 11. Results from linear mixed models (LMM) of male age at maturity with chi-square (χ2) values for significance tests of estimated parameters for diet, temperature, and sex.

| Full model | | | Estimate | *SE* | *df* | χ² | *P* |
| --- | --- | --- | --- | --- | --- | --- | --- |
| Fixed effects | |  |  |  |  |  |  |
| Intercept (control, control, female) | | 49.830 | 0.666 | 129.749 | 5597.141 | < 0.001 |  |
| Diet (restricted) | | 9.335 | 0.881 | 197.898 | 112.145 | **< 0.001** |  |
| Temperature (high) | | -9.963 | 0.910 | 202.770 | 119.925 | **< 0.001** |  |
| Diet * Temp | | 2.713 | 1.269 | 200.280 | 4.568 | **0.033** |  |
| Random effect | **Variance** | ***sd*** | **Number of groups** | |  |  |  |
| Brood ID (intercept) | 0.764 | 0.874 | 28 | |  |  |  |

Supplementary Table 12. Results from estimated marginal means (EEMs) pairwise comparisons among four treatments (diet * temperature) for male age at maturity with t-ratio values for significance tests.

| Contrast | t ratio |  | p value |
| --- | --- | --- | --- |
| Control diet, control temp - Restricted diet, control temp | -10.541 |  | **< 0.001** |
| Control diet, control temp - Control diet, high temp | 10.869 |  | **< 0.001** |
| Control diet, control temp - Restricted diet, high temp | -2.272 |  | 0.108 |
| Restricted diet, control temp - Control diet, high temp | 21.772 |  | **< 0.001** |
| Restricted diet, control temp - Restricted diet, high temp | 8.153 |  | **< 0.001** |
| Control diet, high temp - Restricted diet, high temp | -13.117 |  | **< 0.001** |

Supplementary Table 13. Results from linear mixed models (LMM) of size at maturity with chi-square (χ2) values for significance tests of estimated parameters for diet, temperature, and sex. Given the three-way interaction, this model was divided for males and females (see Supplementary Table 14 and 16).

| Full model | | | Estimate | *SE* | *df* | χ² | | *P* |
| --- | --- | --- | --- | --- | --- | --- | --- | --- |
| Fixed effects | |  |  |  |  | |  |  |
| Intercept (control, control, female) | | 17.021 | 0.167 | 99.591 | 10338.480 | | < 0.001 |  |
| Diet (restricted) | | -0.839 | 0.183 | 394.725 | 21.008 | | **< 0.001** |  |
| Temperature (high) | | 1.195 | 0.182 | 397.620 | 43.103 | | **< 0.001** |  |
| Sex (male) | | -1.244 | 0.186 | 397.985 | 44.811 | | **< 0.001** |  |
| Diet * Temp | | 0.930 | 0.264 | 396.746 | 12.384 | | **< 0.001** |  |
| Diet * Sex | | 0.720 | 0.263 | 398.994 | 7.467 | | **0.006** |  |
| Temp * Sex | | -1.517 | 0.269 | 402.115 | 31.797 | | **< 0.001** |  |
| Diet * Temp * Sex | | -0.886 | 0.382 | 401.144 | 5.389 | | **0.020** |  |
| Random effect | **Variance** | ***sd*** | **Number of groups** | | |  |  |  |
| Brood ID (intercept) | 0.309 | 0.556 | 28 | | |  |  |  |

Supplementary Table 14. Results from linear mixed models (LMM) of female size at maturity with chi-square (χ2) values for significance tests of estimated parameters for diet, temperature, and sex.

| Full model | | | | Estimate | | *SE* | *df* | | χ² | *P* |
| --- | --- | --- | --- | --- | --- | --- | --- | --- | --- | --- |
| Fixed effects | | |  | |  |  | |  |  |  |
| Intercept (control, control, female) | | | 17.008 | | 0.206 | 64.233 | | 6798.934 | < 0.001 |  |
| Diet (restricted) | | | -0.904 | | 0.224 | 194.555 | | 16.330 | < 0.001 |  |
| Temperature (high) | | | 1.143 | | 0.224 | 197.347 | | 26.121 | < 0.001 |  |
| Diet * Temp | | | 0.979 | | 0.324 | 196.164 | | 9.108 | **0.002** |  |
| Random effect | **Variance** | ***sd*** | | **Number of groups** | | |  |  |  |  |
| Brood ID (intercept) | 0.451 | 0.671 | | 28 | | |  |  |  |  |

Supplementary Table 12. Results from estimated marginal means (EEMs) pairwise comparisons among four treatments (diet * temperature) for female size at maturity with t-ratio values for significance tests.

| Contrast | t ratio |  | p value |
| --- | --- | --- | --- |
| Control diet, control temp - Restricted diet, control temp | 4.034 |  | **< 0.001** |
| Control diet, control temp - Control diet, high temp | -5.097 |  | **< 0.001** |
| Control diet, control temp - Restricted diet, high temp | -5.296 |  | **< 0.001** |
| Restricted diet, control temp - Control diet, high temp | -9.031 |  | **< 0.001** |
| Restricted diet, control temp - Restricted diet, high temp | -9.047 |  | **< 0.001** |
| Control diet, high temp - Restricted diet, high temp | -0.32 |  | 0.989 |

Supplementary Table 16. Results from linear mixed models (LMM) of male size at maturity with chi-square (χ2) values for significance tests of estimated parameters for diet, temperature, and sex.

| Full model | | | Estimate | *SE* | *df* | χ² | | *P* |
| --- | --- | --- | --- | --- | --- | --- | --- | --- |
| Fixed effects | |  |  |  |  | |  |  |
| Intercept (control, control, female) | | 15.875 | 0.137 | 58.028 | 13460.200 | | < 0.001 |  |
| Diet (restricted) | | -0.202 | 0.128 | 184.497 | 2.480 | | 0.115 |  |
| Temperature (high) | | -0.392 | 0.134 | 187.031 | 8.623 | | 0.003 |  |
| Diet * Temp | | 0.092 | 0.185 | 185.002 | 0.246 | | 0.620 |  |
| Random effect | **Variance** | ***sd*** | **Number of groups** | | |  |  |  |
| Brood ID (intercept) | 0.264 | 0.513 | 28 | | |  |  |  |
| Reduced model | | | **Estimate** | ***SE*** | ***df*** | **χ²** | | ***P*** |
| Fixed effects | |  |  |  |  | |  |  |
| Intercept (control, control, female) | | 15.851 | 0.128 | 46.280 | 15431.040 | | < 0.001 |  |
| Diet (restricted) | | -0.157 | 0.092 | 186.711 | 2.915 | | 0.088 |  |
| Temperature (high) | | -0.344 | 0.092 | 186.843 | 13.878 | | **< 0.001** |  |
| Random effect | **Variance** | ***sd*** | **Number of groups** | | |  |  |  |
| Brood ID (intercept) | 0.261 | 0.511 | 28 | | |  |  |  |
